# Qualitative exploration of the medical learner’s journey into correctional health care at an academic medical center and its implications for medical education

**DOI:** 10.1007/s10459-020-09997-4

**Published:** 2020-10-19

**Authors:** Ahmar H. Hashmi, Alina M. Bennett, Nadeem N. Tajuddin, Rebecca J. Hester, Jason E. Glenn

**Affiliations:** 1 Department of Family Medicine, Faculty of Medicine, Chiang Mai University, 110 Intharavoros Road, Chiang Mai, 50220 Thailand; 2 NCal Regional Ethics Department, Kaiser Permanente, Northern California, Oakland, CA 94612 USA; 3 Greater Houston Endocrinology, PLLC, Houston, TX 77089 USA; 4 Department of Science, Technology and Society, Virginia Polytechnic Institute and State University, Blacksburg, VA 24061 USA; 5 Department of History and Philosophy of Medicine, Medical Center, University of Kansas, Kansas City, KS 66160 USA

**Keywords:** Academic medical centers, Attitude, Curriculum, Graduate medical education, Prisoners, Standard of care, Undergraduate medical education

## Abstract

Correctional systems in several U.S. states have entered into partnerships with academic medical centers (AMCs) to provide healthcare for persons who are incarcerated. One AMC specializing in the care of incarcerated patients is the University of Texas Medical Branch at Galveston (UTMB), which hosts the only dedicated prison hospital in the U.S. and supplies 80% of the medical care for the entire Texas Department of Criminal Justice (TDCJ). Nearly all medical students and residents at UTMB take part in the care of the incarcerated. This research, through qualitative exploration using focus group discussions, sets out to characterize the correctional care learning environment medical trainees enter. Participants outlined an institutional culture of low prioritization and neglect that dominated the learning environment in the prison hospital, resulting in treatment of the incarcerated as second-class patients. Medical learners pointed to delays in care, both within the prison hospital and within the TDCJ system, where diagnostic, laboratory, and medical procedures were delivered to incarcerated patients at a lower priority compared to free-world patients. Medical learners elaborated further on ethical issues that included the moral judgment of those who are incarcerated, bias in clinical decision making, and concerns for patient autonomy. Medical learners were left to grapple with complex challenges like the problem of dual loyalties without opportunities to critically reflect upon what they experienced. This study finds that, without specific vulnerable populations training for both trainees and correctional care faculty to address these institutional dynamics, AMCs risk replicating a system of exploitation and neglect of incarcerated patients and thereby exacerbating health inequities.

## Introduction

Currently, there are a handful of different models for delivering secondary and tertiary healthcare to incarcerated populations (US Department of Justice & National Institute of Corrections 2001), with most correctional healthcare delivered via contracts with private, for-profit companies. Private correctional healthcare companies lack public accountability and governmental oversight and their primary fiduciary responsibility is to shareholders and profit margins, not to the patients (Andrews 2017). This has resulted in the horrific and systemic neglect of private, for-profit healthcare delivered at both public and private prisons (Wilper et al. 2009; Neate 2016). Having academic medical centers (AMCs) provide healthcare for incarcerated persons could theoretically mitigate such problems, with AMCs now representing the second most commonly outsourced system through which incarcerated persons receive healthcare (Min et al. 2012).

AMCs have justified their involvement in correctional care as being uniquely placed to improve population health and address health disparities among those incarcerated (Baillargeon et al. 2000, 2004; Bick 2007; Raimer et al. 2010). In addition to these reasons for AMC involvement in the provision of correctional health care, proponents of academic-correctional health partnerships claim that correctional care environments provide fertile training grounds for medical graduates and undergraduates. A 2012 review found 22 U.S. academic medical programs that provided highly variable levels of correctional health exposure to medical students and/or residents, ranging from brief rotations to clinical fellowships in correctional health—with little to no mention of ethics or vulnerable populations training included as part of an academic program’s correctional health curriculum (Min et al. 2012).

In extolling the virtues of training with patients who are incarcerated, AMCs and correctional care programs assume that exposure to incarcerated populations leads to positive changes in attitudes and behaviors of the medical learner that may be enhanced through structured training. This assumption is ostensibly founded on studies investigating attitudes of health professionals working in corrections, supported by assessing said attitudes through use of scales and indices such as Attitudes Toward Prisoners (ATP), Attitudes Toward Mentally-Ill Offenders, or the Interpersonal Reactivity Index (Melvin et al. 1985; Dhawan et al. 2007; Kjelsberg et al. 2007; Church III et al. 2009). These studies posit that “proximity” to the incarcerated results in more positive attitudes (Melvin et al. 1985; Kjelsberg et al. 2007), as does higher education (Church III et al. 2009), and associate exposure to patients that are incarcerated with greater empathy and compassion (Dhawan et al. 2007). A few AMCs offering curricula or training with vulnerable populations point to the positive impacts of early educational experiences for health professional trainees in correctional environments as well as for trainees working with the underserved, communities of lower socioeconomic status, and minority populations (Kaufman et al. 1979; Alemagno et al. 2004; Littlewood et al. 2005; Kuthy et al. 2007). Some studies suggest that exposure to the incarcerated or underserved can engender a willingness to serve such populations in the future (Kuthy et al. 2007; Brooker et al. 2018).

However, these studies are often limited by methodology or the outcomes analyzed. Oftentimes, the use of validated scales to measure attitudes toward incarcerated persons or the vulnerable is limited by cross-sectional designs. Exploratory studies employ mixed-methods but are limited by analytical frameworks focused on benefits to the trainee or their willingness to serve the vulnerable in the future. Without longitudinal assessments of exposure to the vulnerable or curricula to engage learners around issues of vulnerability, however, the studies highlighted here do little more than suggest immediate and short-term benefits for trainees.

Therefore, curricula designed to promote competencies in correctional care would be better informed through a more robust exploration of the challenges and difficulties medical trainees experience absent such a curricula. With the largest prison population in the U.S. at roughly 150,000, the Texas Department of Criminal Justice’s (TDCJ) capitated managed care contract with the University of Texas Medical Branch (UTMB) is recognized as a national leader for the academic-correctional partnership model (Raimer and Stobo 2004). UTMB is the only AMC to have a free-standing hospital dedicated to providing tertiary-level care to incarcerated patients. Medical students and residents at UTMB routinely treat patients in the “TDC Hospital,” supervised by attending physicians, as part of their training and education. Hence, the unique setting that UTMB provides allows a fuller, more open-ended exploration of medical learners’ perspectives on training and providing healthcare services to incarcerated patients where other studies may be lacking. The purpose of this study is to characterize the attitudes and perceptions of medical students and medical residents toward training in a correctional care environment.

### The health of those who are incarcerated

The healthcare delivered to persons who are incarcerated has long been recognized as below the standard of care that is delivered to free-world populations (Wilper et al. 2009; Fazel and Baillargeon 2011; Borysova et al. 2012). As the greatest determinant of an incarcerated person’s health, the prison represents a “crucible” within which numerous risk factors and exposures converge, such as trauma-inducing violence (Freudenberg 2001), crowded living conditions, poor sanitation and ventilation (Bick 2007; Awofeso 2010), and a lack of healthy dietary or exercise options in prison (Baillargeon et al. 2000). Prior to incarceration, incarcerated individuals often hail from communities with limited access to adequate primary care. These vulnerabilities, both prior to and during incarceration, are reflected in the high burden of infectious diseases among the incarcerated, such as HIV, tuberculosis, hepatitis B and C. A trend toward lengthier sentences further increases the chronic disease burden in this population (Baillargeon et al. 2000, 2004; Bick 2007; Raimer et al. 2010). In addition, more than half of the incarcerated have a mental health problem, including high rates of substance use disorders (US Department of Justice & Bureau of Justice Statistics 2006; Baillargeon et al. 2009; Prins 2014; Fazel et al. 2017).

The burden of disease varies when broken down by race. Within the TDCJ, the age-adjusted prevalence of chronic medical conditions among Blacks was greater than that of Whites for hypertension, asthma, and diabetes but lower for ischemic heart disease (Harzke et al. 2010). Although diabetes prevalence among Hispanics was similar to that of Blacks, Hispanics had a lower prevalence of chronic conditions such as hypertension, ischemic heart disease, cerebrovascular disease, asthma, and chronic obstructive pulmonary disease (Harzke et al. 2010). An earlier study showed that White men had lower rates of tuberculosis than Black or Hispanic men, but higher rates of viral hepatitis; Black men had the highest rates of HIV/AIDS, followed by Hispanic men (Baillargeon et al. 2000). When considering gender, most incarcerated women are in their thirties and have minor children, and are often imprisoned for nonviolent drug and property crimes (Bloom and Covington 2008). Prior to their incarceration, these women are medically underserved, many have not seen a health care provider in the past year, and they have limited health care access before arriving in jail or prison (Conklin et al. 2000). Consequently, when compared to men, women who enter jail are more likely to have a history of homelessness, illicit drug use, and multiple health problems (Freudenberg 2001). Women arrive at and leave prison with an increased incidence of undertreated chronic health problems, including asthma, hypertension, heart disease, and diabetes (National Commission on Correctional Care 2002; Meave 2003). Histories of substance use, multiple sexual partners, and inconsistent contraceptive use also place women at high risk for unplanned pregnancies and sexually transmitted infections, including HIV and Hepatitis B and C viruses (Clarke et al. 2006). Incarcerated women also report more frequent histories of childhood and adult violence, including physical and sexual assault (Browne et al. 1999; Fickenscher et al. 2001). Finally, rates of psychiatric disorders are higher among incarcerated women than among incarcerated men. They report higher rates of mania, major depression, anxiety, post-traumatic stress, personality, psychotic, and substance use disorders (Abram et al. 2003; Covington 2007).

## Methods

This study employed a qualitative, exploratory approach, conducting focus group discussions (FGDs) with medical students in the School of Medicine and medical residents in the Internal Medicine Residency Program at UTMB, Galveston, Texas, USA.

The research team was made up of UTMB medical students (AH, NT), a UTMB student completing a PHD in Medical Humanities and a MPH (AB), and two professors from the UTMB Institute of Medical Humanities (JG, RH). All FGDs were facilitated by at least one member of the research team (AH or AB). This was by design such that FGD participants would perceive facilitators more as peers than as superiors. Hence, as a fourth year medical student, AH led FGDs with medical students, while AB as a graduate student (neither medical resident nor medical student) led FGDs with residents with AH or AB serving as a facilitator for the other.

Semi-structured FGD guides were developed by investigators AB, AH, and NT and revised by JG and RH. This short guide aimed to qualitatively explore attitudes toward the incarcerated, loosely based on the Attitudes Toward Prisoners scale (Melvin et al. 1985). The guide also incorporated findings from a review of literature related to medical and other health professional trainee experiences with incarcerated persons and other vulnerable populations (Alemagno et al. 2004; Kjelsberg et al. 2007; Kuthy et al. 2007). These guides were further informed by the clinical and educational experiences of AB, AH, NT, and JG in the TDC Hospital. The main areas for discussion included: initial impressions about providing care in TDC Hospital; experiences and feedback on the TDC Hospital orientation; thoughts about practicing on incarcerated patients for medical training or research purposes; the overall purpose of the prison system; curiosity about an incarcerated person’s conviction and potential impact on care; and how the TDC Hospital rotation supported and/or challenged professional and personal values. Discussion guides were developed to allow a maximum one-hour discussion with participants.

FGDs were conducted between December 2014 and February 2015. The research team worked with course coordinators in identifying a list of potential medical trainees from the School of Medicine and the Internal Medicine Residency Program. Aside from a group of medical students in their first year who were selected at random from the existing roster, medical students in their third and fourth years of training and internal medicine residents who had completed at least one, one-month rotation in TDC Hospital were selected at random. This allowed inclusion of participants who had and had not completed a formal clinical rotation in TDC Hospital. By study end, 8 FGDs were conducted with a total of 30 participants including medical students (MS I, MS III, MS IV) and residents (PGY-1, PGY-2, PGY-3) (Table 1). Women made up 60.0% (18/30) of all participants, with medical students ranging from 20 to 28 years of age and medical residents ranging from 25 to 35 years of age.

**Table 1:** Participant demographics (MS: medical student; PGY: post-graduate year, Internal Medicine residents)

Trainee Level	Women	Men	Total
MS I	7	3	10
MS III	3	2	5
MS IV	1	1	2
PGY-1	3	0	3
PGY-2	4	2	6
PGY-3	0	4	4
Total	18	12	30

To allow for greater discussion and limit “power differentials” between participants at different levels of training, groups were purposively selected such that each group had participants at the same level of training. FGDs were conducted in private rooms on the UTMB campus, outside the TDC Hospital. Participants were given participant information sheets outlining risks and benefits of the study, and facilitators introduced themselves according to their position within their respective schools. After participants provided signed informed consent, facilitators began audio recording and noted participant age and gender. No other identifying information was collected. Facilitators attempted to use participant names minimally and participants were instructed to do the same to preserve anonymity.

Notes by both facilitators were compiled following each FGD and reviewed by lead investigators (AH and AB). Facilitators debriefed after FGDs with the intent to determine if additional FGDs were needed. Following FGDs, audio recordings were uploaded and kept on a password-secure computer. Audio recordings were transcribed verbatim and transcripts were further password-protected.

### Analysis

The most pertinent ontological grounding that informed this research is a constructivist paradigm as described by Guba and Lincoln (1994). Analysis followed a subjective inductive approach (Varpio et al. 2019), informed by a conceptual framework put forth by Genn where, “the educational climate is a manifestation or operationalization of the educational environment and of the curriculum” (Genn 2001). Over the course of inductive analysis, this conceptual framework was adjusted to align with themes emerging from trainee narratives. We attempted to speak truth to the trainees’ collective voice who conceived of a “culture of low prioritization and neglect” as manifested by the learning environment they encountered while on correctional care rotations in the TDC Hospital. Hence, we summarize our results in a specific pattern: allowing the participants to describe this culture of low prioritization and recording their lived experiences within this broader culture over the course of their correctional care rotations.

Three investigators (AH, JG, and AB) performed preliminary analysis on FGD transcripts using NVivo 12, NVivo for Mac, or Microsoft Excel and Microsoft Word according to their preference. All investigators did line-by-line inductive analysis. Codebooks from three investigators were compiled separately, then repeated discussions were held to finalize codebooks, after which individual investigators then re-analyzed transcripts according to these codes. Upon completion of each step of analysis, AH compiled results in one master file using NVivo for Mac. Inter-reliability analysis was performed using this master file to identify discrepant coding interpretations for further clarification between investigators as needed. Individual investigators performed thematic analysis as a final phase, which was then discussed and compiled. This process allowed for saturation, triangulation, and corroboration of main findings.

## Results

Participants depicted a prevailing institutional culture of low prioritization and neglect that encompassed both structural barriers and institutional factors, established by attending physicians, nursing staff, and corrections officers, that resulted in the delivery of substandard care to the incarcerated. Having established the bounds of this learning environment within the correctional care complex, the medical trainees reflected on their experiences in correctional care by discussing their preconceptions prior to clinical rotations; their orientation to the TDC Hospital; the moral and ethical dimensions of providing care to the incarcerated; their struggle with perceived dual obligations, both to the patients and to the corrections system; and ways in which the correctional care experience challenged their preconceived notions of those who are incarcerated.

### Low priority patients

As participants described the learning environment at length, a central theme emerged: how an institutional culture of low prioritization and neglect, combined with structural barriers within the TDC Hospital and the greater prison health complex, often resulted in poor health outcomes for the incarcerated. This culture of low prioritization and neglect centered around a prevailing sense of the “unworthiness” of those who are incarcerated. Participants picked up on the implicit and explicit expression of this attitude in attending physicians, senior nurses, and consultant services on the wards, and in institutional policies that prioritized the diagnostics, lab work and procedures of free-world patients over those for incarcerated patients. As this prevailing culture of low prioritization and neglect collided with structural barriers inherent to the prison health complex, participants linked both to the resultant delayed substandard care for the imprisoned. The following sub-themes highlight these elements that make up the learning environment in correctional care at the TDC Hospital.

#### Health care staff attitudes toward the incarcerated patients

Analysis of FGDs revealed that health care staff within the TDC Hospital frequently perpetuated negative moral judgments and mistrust of incarcerated patients. Medical learners noted many instances where correctional care staff—including attending physicians and nurses—espoused such views resulting in a markedly less empathic response to incarcerated patients.

“I can think of people I've known who comment on care for prisoners, thinking of whatever crime they committed and think they don't deserve anything. It’s a viewpoint of a lot of people I know.”—MS I.

"[W]e had attendings that [sic] felt that way. When patients would say they're in pain [some attendings would be] like, ‘Oh, well, they're a criminal, they did something to get here. They're a professional liar,’ and stuff like that. So they didn't believe they were really having pain. And sometimes they might be right but I know they wouldn't do that with a free world patient. Because a free world patient could be a criminal, they could have gotten out of jail last week and you're not going to ask every patient about their criminal history before you give them pain medication."—MS III.

Here we see that a culture of neglect espoused by attending physicians relates to overarching views toward justice-involved persons held by the general public, and that these views lead to bias–implicit and explicit–among those entrusted to provide care to the incarcerated. This bias crept into the day-to-day care of patients at TDC Hospital, as a second year medical resident connected the perceived burden of making the trek to the TDC Hospital to a prevailing belief in the “unworthiness” of the imprisoned:

“[Y]ou can even see it in the faculty, [who say] ‘Aw yeah, we can do table rounds [i.e., discussing a patient’s care on a given day without physically seeing the patient]—I don't really need to go over there.’ That happens.[…] Because TDC [patients] are looked at as lesser people than our free world [patients…]. That may not be how we see it but I think that's probably the truth.”—PGY-2.

Another manner in which attending physicians perpetuated the perception of unworthiness of incarcerated patients was by encouraging their moral judgment among learners. Many physicians modeled this behavior by researching a patient’s criminal record prior to each encounter in the TDC hospital. In Texas, as in many other states, a person’s criminal record is accessible to the public. Participants pointed out how attending physicians helped normalize researching the crime for which incarcerated patients were convicted, and at times directly informed the medical team.

“I had an attending on [a specialty service] who would Google it before we went into every room.[…] He would Google them, every time we went into a room, to see what they did. I'm not sure why.”—MS III.

Trainees reported receiving the same mentored guidance from correctional nursing staff. One medical student provided a clear example of the ways in which providing compassionate care to an incarcerated patient could be hampered by this practice, coercing members of the healthcare team to adopt a more penal orientation toward incarcerated patients. He reported on the warnings he would receive from correctional nursing staff before entering patient rooms:

“‘Oh he’s a serial rapist that you’re going [to see] in there. And he has erectile dysfunction and that’s what you get’ and blah blah blah. They would have these opinions about why [we shouldn’t] feel bad for him. I would come out and say, ‘Oh man, I feel bad for this guy, he’s got no legs and he’s got pain and this chronic pain from a double amputation.’ And they’re like, ‘Don’t feel bad for him he raped six boys.’ I still feel bad for him but now I honestly don’t feel as bad for him.”—MS III.

This overarching culture of low prioritization underlined the experiences medical trainees had while learning within the confines of the TDC Hospital. Informed by societal stereotypes about justice-involved persons, redolent with bias, represented across multidisciplinary healthcare teams, and serving to impune the character and disparage the needs of the incarcerated patient, this culture plays a central role in perpetuating beliefs about the unworthiness of the incarcerated that impedes responsive health care.

#### Structural barriers and resultant delays in care

In addition to this broader culture of neglect underpinned by the “unworthiness” of patients, participants illustrated the ways in which structural barriers—both within the TDCJ Hospital as well as in the prisons throughout the state that TDC patients come from–delayed access to diagnosis and treatment and limited the continuity of care. This resulted in worsening conditions, situations requiring emergent care, and poor health outcomes for incarcerated patients. For the participants, these structural barriers to care began on the home unit:

“I think a lot of times when they go to their unit doctor, the doctors dismiss them. By the time they actually get brought to the hospital it’s really shocking.”—MS III.

Should patients reach the TDC Hospital in spite of this “gatekeeping” at their home units, these delays in care could be further perpetuated while in the TDC Hospital. Participants often reflected how the routine care, diagnostics, and treatment provided at the TDC Hospital were readily superseded by the competing needs of UTMB’s “free world” patients receiving care in the adjacent hospital.

“All I could think of was, 'Don't code in TDC.’ You know, that sounds terrible but it’s just an access issue.”

Many participants related to the structure of the built environment within the TDC Hospital, reflecting the prison systems’ prioritization of safety and security.

“MRIs [take longer] because they need to take officers, they have to get another officer, they have to transport them down. Regular labs—I don't know if maybe this has to do with the nursing—but they kind of put things off; if you're on back float and admit a patient at 7:00 PM they'll wait to do labs until morning. So we've noticed that too, in terms of getting stuff done it takes a lot longer at TDC.”—PGY-3 FGD.

“It’s also not good for the patients. I specifically remember one instance where my patient was in acute pain and I made sure I told the nurse to get him some morphine. Three hours later, I come around and he's still in pain and hasn't gotten his morphine and she says, ‘Well I couldn't get a guard to supervise me,’ so then the patients don't get what they need.[…] It’s not our fault, they just can't get what they need sometimes.”—PGY-3 FGD.

In addition to these logistical challenges in the TDC Hospital environment, the built environment of the TDCJ health care apparatus often limited access to medications, treatments, and care facilities, as medical trainees pointed out:

“[T]he number one thing that prevents good care of these patients is not the personal biases towards inmates but rather the limitations of the system. What we can and cannot do for these patients.[…] They have very specific lists of medicines and sometimes there’s not a class you can prescribe for that medicine. There’s no alternative. You *have* to get this or you get this problem untreated. I mean that’s a far bigger—far bigger—issue when it comes to treating these patients than the patients themselves.”—MS III.

Even discharging TDC patients often provided additional delays that sometimes affected care.

“[B]arriers to discharge—there are definitely those in the TDC [Hospital]. It would be the equivalent of social work issues like finding a unit that will take them [upon discharge].[…] [I]t’s not like the prison system is not very flexible in its ability to accommodate—it’s just that they can't. There are not enough beds at these locations. You just accept that as part of the barriers to discharge.”—MS IV.

In summary, medical trainees depicted the nature of their learning environment, where the combination of the culture of neglect and the structural issues within the TDC Hospital and the greater prison health complex often resulted in poor health outcomes on the part of the incarcerated.

### The medical learner’s journey into correctional care

Having expounded upon the learning environment of a correctional care rotation within the TDC Hospital, we asked medical trainees to describe the lived experience of training in this setting. Participants expressed their feelings prior to performing their clinical duties, considered their preparedness upon entering this environment, and reflected on key topics such as personhood of the incarcerated, conflicts between the goals of medicine and those of the correctional establishment (“dual loyalty”), and positive outcomes from training in this environment. As expected, these discussions focused on what it was like to be a medical learner against the backdrop of the prevailing culture of low prioritization and neglect, along with the structural barriers inherent to the TDCJ health complex.

#### Preconceptions of caring for incarcerated patients

Prior to beginning rotations in the TDC, many participants had at least heard of the possibility that they may perform rotations with incarcerated patients within a high security facility. Most medical learner concerns hinged on the “fear of the unknown” in terms of how their security would be ensured while caring for incarcerated patients:

“All these thoughts come in your mind of, ‘I'm going to be a third year going into a little room with someone…’ You know what I mean? Then you hear a horror story of someone swallowing a knife and trying to use it to kill [their doctor]. No one is telling you what the day by day is like so the only thing you have is what you previously came in with, for those of us with no experience of that whatsoever it was just a little scary.”—MS I FGD.

Some of these fears were the result of misconceptions of those who are incarcerated, often reflecting stereotypes that the majority of criminals were violent offenders, as well as how easily these stereotypes could be perpetuated among trainees:

“Yeah, because they're prisoners and [have] a lot of crime history, and anybody could have committed a violent crime or they could be murderers or rapists, so I'm really scared because those people could be more aggressive. Or [thinking,] ‘How am I going to work with them?’”—PGY-3.

The manner in which UTMB informed newly admitted trainees of these rotations— by way of having students provide signed disclosures prior to matriculating to UTMB—often exacerbated these fears:

“[A]s part of our initial information packet we had to fill out all these release forms and I remember being in shock because it was the longest [form]. It was about a 4-page form and we had to disclose if we had any relationship with gangs or tattoos or if we had any family members in the TDCJ. So that was interesting, how much they wanted to know—I guess to protect us and protect them.”—MS I FGD.

#### Orientation to the TDC Hospital

As with most clinical rotations in undergraduate and graduate medical programs, a TDC Hospital orientation is implemented as part of the UTMB program. Medical trainees noted that UTMB provides no specific training on the care of vulnerable populations or in ethical issues relating to correctional care. Instead, the provided orientation predominantly focused on security and safety:

“First time they tell you, you have to be careful—they give you security measures, what to do. They will tell you what kind of information you can repeat to the patients.”—PGY-3.

The orientation also highlighted health service delivery and statistics, often lost on the trainees:

“It was just a bunch of statistics like, ‘There are this many infirmary beds, this many hospitals, and this many inmates in the TDC system,’ and you get this idea of very limited resources, basically. He went through all the statistics of that and what the turnover rate has to be in order for it to be viable. So it was a lot of numerical—like crunching the numbers on how many patients we serve, how many beds there are, and limited resources and this and that.”—MS III.

Furthermore, the timing and frequency of the training was generally inconsistent, with medical residents receiving the training multiple times whereas medical students often received their orientations *after* beginning their rotation or sometimes, not at all. This orientation—or lack thereof—left participants both unaware and unprepared for training in correctional care.

Hence, we find that the orientation for medical trainees to the TDC Hospital service terribly lacking. An opportunity to assuage trainee fears and anxieties, this orientation seems instead to have potentially stoked them. It is a lost opportunity to engender a more holistic understanding of justice-involved persons that combats the very stereotypes that are perpetuated by the prevailing culture of neglect. Furthermore this orientation did little to equip medical trainees for the morally and ethically precarious positions they were to find themselves in while immersed in the prison health complex over the course of their clinical rotation.

#### The ethical and moral dimensions of correctional care and the specific vulnerabilities of the incarcerated

“We kind of forget that incarcerated people are human beings. They have personalities, they have loves and interests and passions.”—MS I.

As one may predict, the culture of low prioritization and neglect, with its explicit and implied pervasive moral judgments about incarcerated patients, crept into the psyche of medical trainees—especially the younger medical students. Briefly, the ethical and moral dimensions of being exposed to correctional care were complex. Although many topics were broached, in general medical trainees failed to acknowledge that the special vulnerability of being incarcerated often curtails patient autonomy, and that bias only worsens the already precarious position of the incarcerated.

Therefore, without structured guidance on such important issues, we begin to see medical learner’s perceptions and actions reflect the perception of incarcerated patients as unworthy of their best efforts, as espoused by their mentors. This included a general distrust of patient complaints and the assumption of malingering; curiosity about an incarcerated individuals’ conviction and its relation to biasing patient care; and the security apparatus dampening a trainees’ willingness to round in the TDC Hospital. In particular, the assumption of malingering and manipulation among the incarcerated were both influenced by, and directly influenced, participants’ beliefs about their patients:

“Yes, because they are prisoners sometimes they think like they’re faking. That is a main factor—ignorance. Everybody puts the same label on them that they're faking and in reality some of them really pay for it—those who are really sick. They are not able to get immediate medical care. *So we cannot really blame anybody because the population they are dealing with.*”—PGY-3 [emphasis added].

“Generally, I feel like if someone says something like [patients are manipulative or malingering]…We definitely have patients [like that]—but it’s not that bad. But normally [residents who believe that a patient is malingering] have reasons, like they can back it up, ‘No, they were totally fake limping while doing the [neurological] exam.’ There’s a fact that will back up whatever they’re saying. *So it might be a population that might be more prone to being manipulative*. We talk a lot about ‘frontal lobe pathologies’ [laughter]. Because we have a [Neurology] resident on our team. But also we don’t just toss out the notion that, ‘No, actually they don’t feel pain.’ Our team deals a lot with pain. We really don’t just brush off this notion of patients being in real pain.”—MS III, [emphasis added].

The custom of seasoned correctional health staff researching the crime for which an incarcerated patient was convicted was often adopted by medical learners. Participants—especially the medical students—had difficulty navigating the ethical challenge of whether to look up a patient’s record. Some could see how doing so might introduce bias into their clinical decision making:

“I wouldn't [look up a patient’s criminal record] because I feel it would affect the way I see the person and I wouldn't want that… If I know what they did and it’s something that I felt [sic] strongly about, I may not even do it on purpose, but I may not do the hardest that I can.”—MS I.

Although acknowledging that one’s curiosity could compromise the quality of the care provided, the majority of medical learners viewed this choice to be the prerogative of the care provider in managing their newfound knowledge about a given incarcerated patient:

“Whether you act on that curiosity or not depends on the person, largely. And to take it a step further—does it impact your care? I would say, ‘No.’ I would never see it impact a patient’s care before.”—MS III.

As we saw above, attending physicians bore their own biases against the incarcerated by a reluctance to even round and see them in person on the wards. Citing delays within the TDC hospital in navigating security measures, trainees checked on their incarcerated patients less frequently. Although not explicitly linked to bias against justice-involved persons, it strikes us that the culture of neglect permits this reluctance to check on their incarcerated patients as frequently as trainees do their “free-world” counterparts.

“I do have an issue with the ICU care there [in the TDC Hospital]… [I]t’s hard because we take care of the free world critical and TDC ICU at the same time. That creates a challenge because we have so many more patients [in the TDC Hospital]. They do have a nurse practitioner there … all the time but it’s a long walk so that part makes it harder to take care of [TDC] patients when you're mainly based on [the free world] side. You can't see them as often.”—PGY-1.

“You're just unwilling to go see them. Sometimes at the university hospital, sometimes I want to check on the patient more often. So I don't need any handlers…But if I have to wait long, maybe subconsciously I would avoid checking more.”—PGY-3.

In these subtle—and often, unconscious—ways, medical trainees were now perpetuating elements of the culture of neglect that permits second-rate care for the incarcerated.

Trainees described how they were granted greater leeway by attendings to practice their skills on incarcerated patients, citing incarcerated patients’ lack of social support from friends or family members advocating for their care, and noted the lack of institutional pressure mitigating patient risks in diagnosis and treatment. Surprisingly, these were viewed less as an ethical concern infringing upon patient autonomy and respect for persons, and more of a benefit of training within the prison health complex.

“Free-world people don’t like getting rectal exams from students. TDC patients have no choice or they don’t care. And to me that was a big advantage—I mean, not that I’m really fond of rectal exams—but it was a good learning experience to be able to do these exams with no pressure of, ‘If you mess this up, they’re going to complain to the hospital or complain to the attending,’ or something like that. There’s no threat of um, y’know, retribution or whatever. Repercussions.”—MS III.

“You discover that it’s heaven there [group laughter]. There's a lot of social issues in the free world that you don't get involved with in TDC. You spend less time talking to families and people trying to intervene in your management and direct you on what to do because usually the prisoners will agree to go the way you want. You would explain everything and get their informed consent but in the real world they would ask for a second opinion and argue with you and they have doctors from outside that try to jump in the picture and dictate what to do. These issues you don't find in the TDC setting.”—PGY-3.

Unbeknownst to the participants, their description of being able “to do more” with TDC patients captures just how ingrained this culture of neglect truly is—mediated by the obviation of incarcerated patients’ personhood and the unconscious exploitation of the precarious nature of being a justice-involved person.

Another element lost on these medical learners was how an incarcerated patient’s specific vulnerabilities were inextricably linked to the clinical pathology observed. Indeed, many of the medical students and particularly the medical residents were enamored with the TDC Hospital upon discovering the advanced clinical pathology suffered by incarcerated patients.

“[I]t was one of the drawing factors, when I was interviewing here for UTMB I thought ‘Oh, I don't want to come to Galveston.’ I was going to use this place as a practice interview but then I came here, I loved the program, the opportunities with the TDC—and I'm interested in doing infectious disease so the TDC provides a lot of great pathology. Like all the fungal infections, stuff I wouldn't see at my medical school[…]. Here, my first work month was my first month of residency and I saw streptococcal meningitis, histoplasmosis, TB—very commonplace… it was kind of a drawing factor to TDC for me personally.”—PGY-3.

Participants, however, could still forge a more explicit link between moral judgment of the incarcerated as persons and the perceived “unworthiness” of incarcerated patients as individuals deserving of medical care. Participants reflected that the aim of their duties was to care for the incarcerated as an individual patient, in spite of the prevailing sense of value (or lack thereof) placed on those who are incarcerated. Frequently and emphatically, medical trainees often asserted an incarcerated patient’s right to care and reiterated the need to look past whatever moral judgment may be placed on the incarcerated in an effort to provide quality care.

"One way you could look at it is—he did something horrible enough to end up on death row. Some people would think he didn't deserve any of the treatment [because of] this. Which is one way of seeing it. I would still treat them, I wouldn't be angry about having to treat them. I would be more saddened to think about all the possibilities that could have led him to death row. Not necessarily the aftermath."—MS I.

Participant 1: “They've already been judged if they're in jail—somebody's already judged them and decided this is their sentence so it’s not really any of my business to do that any further.

Facilitator: To further pass judgment?

Participant 1: Right. Or treat them differently based on that. Because they're already in prison and I'm sure there are people who are not in prison who just didn't get caught doing what they did and so I'm not going to think that about every patient I see out of prison, ‘Did they deserve my care?’ That's not really my call to make.”—MS III FGD.

To summarize, the ethical and moral dimensions presented to medical learners in being exposed to correctional care were complex. What was most concerning about these discussions with medical learners was their lack of sophistication in understanding important ethical implications of providing care to the incarcerated and the broader social forces that shape that care. Although many topics were broached, medical trainees failed to consider that the special vulnerability of incarcerated patients often curtails their autonomy, and that bias only worsens the already precarious position of the incarcerated.

#### Reflections: the dual loyalty of the medical learner in the prison health complex

A significant dilemma for the medical learner emerged from these discussions: how do medical trainees view themselves in the prison health complex when the goals of corrections and medicine are often in conflict? This correctional care experience exposed medical learners to the “dual loyalty” that medical practitioners in correctional settings often face: the conflict between professional duties to a patient and obligations—expressed or implied—by the interests of a third party such as an employer, insurer, or the state (Pont et al. 2012). For example, medical providers treating incarcerated patients cannot promise confidentiality, may not be able to deliver the standard of care as their diagnosis indicates, and can be greatly impeded in consulting family members to aid in decision making.

To determine the depth of medical trainee conceptualization of dual loyalty, facilitators probed for experiences with death row patients, as this often presented a real experience many medical trainees had in addition to presenting an inherent moral challenge. This quote exemplifies the type of ethical and moral struggles medical trainees face while working with the incarcerated:

“Talking about values—with death row patients that was really hard for me because it was like, ‘Wait, you're asking me to help extend their life when you're going to execute them?’ That was hard for me. I had one patient who was on death row and had terminal cancer and I thought, ‘I don't know how this is going to work.’ I actually asked questions like, ‘How aggressive are we going to be?’ Because you're planning on ending his life at some point. That was the only time I've felt like I didn't know what I was supposed to do.”—PGY-1.

Another point of departure—often broached by the participants themselves—provided an example of when providing care was at odds with punishment: the presence of correctional officers. Often, correctional officers’ concerns superseded medical decision-making, in setting up an alternate hierarchy in which the medical team relinquished control to ensure “security.” A clear example of this was how the prison context infringed upon patient confidentiality.

Medical trainees discussed experiences where the correctional officers would make a show of the primacy of “punishment” over “care” in the prison hospital, using subtle ways to establish their superiority over the medical team.

“[The corrections officers can be] passive-aggressive. They won't pay attention to you even though they're not doing anything. One time one of the residents was standing in front of the room and the security was watching her so she said, ‘I'm waiting to see the patient—I’m not paying you to read. I don't stand here for fun, I'm here to see the patient, open the door.’ So you have to speak up—if you stand and they're talking then how will you know? Some of them are really annoying and they obstruct the care sometimes.”—PGY3.

As with the myriad moral and ethical issues medical learners were ill-equipped emotionally and/or psychologically to manage, the issues presented by the dual loyalty inherent in providing care to the incarcerated, the structures of the correctional health setting, and the learners’ status within the medical hierarchy often left them feeling powerless to enact change. In the end, medical trainees often rationalized away their own position in the greater prison health complex.

Participant 1: I feel like we're sidebars. We fix them and send them back to whatever they were doing prior to being patients. I don't necessarily feel like I'm part of that system.

Participant 2: I agree. I'd rather think of it that way anyway. I don't want to be a part of that, I want to be a part of just the sidebars where I do my job and just leave everything else alone.”—PGY-1 FGD.

#### Reflections: positive impacts of correctional care rotations

In spite of ethical and moral issues complicating the care of the incarcerated—those known or unknown to the participants—medical learners often confirmed one clear lesson from working within the TDC Hospital: their previously held beliefs, stereotypes, and fears about those who are incarcerated were challenged by a rotation where participants had to intimately consider incarcerated patients as persons.

“It has provided me with a different perspective of who the prisoners are. As we were saying all along, sometimes they do things and get caught that any other ordinary citizen would do and not get caught and that's why they're in there and still need to be taken care of. Some of them did horrible things but they're still human beings and we need to take care of them. My views of the prison system is way different than before and I feel more comfortable taking care of them.”—MS IV.

"As physicians too though—whether it’s an incarcerated population or not—the skill we're learning to acquire is learning to let go of your pre-judgment. As she said, we all have different backgrounds, upbringings, and we're all going to come into treating incarcerated populations differently based on where we've been, what we've seen, what we've heard. As a physician you have to get rid of those beliefs and stereotypes when you walk into a room because each person is equally important in that moment so you have to do whatever's within you to help that person. Whether it’s emotionally, mentally, physically; that's it. You have to leave all that outside."—MS I.

In balancing perceived conflict between the goals of imprisonment and medicine, medical learners suggested “pushing back” against stigma as a direct result of their experience caring for the incarcerated.

"I would probably try to remove the stigma and stereotype that all prisoners are bad. For people [i.e., other medical trainees] who are especially apprehensive [of providing care to prisoners], just tell them—they weren't any worse or better than the free world patients. They were just in a different part of the hospital is all."—MS III.

Some medical trainees went so far as to promise providing better mentorship through conscious action during the later years of their medical training.

“Because people are going to look up to me next year—they already do. I have people that come to me with questions that I have a responsibility to answer.[…] If I was treated a certain way I want to make sure that I don't do that [to someone else]. So if I see upper levels of mine doing things that I don't agree with, I make a conscious effort to say ‘I should not be doing that next year.’[…] There are always one or two things you disagree with, it’s those things that motivate me to say, 'Next year, I won't do that’—it’s just a responsibility thing. You have to set a good example for other people. You have to do the right thing so other people follow—it’s a ripple effect.”—PGY-1.

## Discussion

This study documents the lived experience of medical learners in a correctional care environment. This exploration provides insight and nuance that both deepens and challenges the discussion around education in a correctional environment that currently exists in the literature. We uncovered the existence of a separate culture of correctional care that permeates the institution, one that perpetuates notions of the “unworthiness” of incarcerated patients to receive the same standard of care as free-world patients. Respondents also illuminated how the built environment of the prison health complex acts as an important structural determinant of an incarcerated patient’s health. Together, these forces contribute to the resultant poor health outcomes consistently witnessed for people who are incarcerated. Medical trainees, thrust into such a training environment and left to grapple with the moral dimensions of these experiences without guidance, struggled to navigate an ethical path.

This study corroborates key findings of other studies in the published literature. It is commonly reported that medical learners experience anxiety and fear prior to performing clinical rotations in correctional care settings, due largely to previously-held cultural biases that those who are incarcerated are disproportionally violent (Alemagno et al. 2004; Weiskopf 2005; Kuthy et al. 2007; Church III et al. 2009; Brooker et al. 2018). However, as in this study, participants’ fears are allayed after exposure to this novel learning environment. Implications for the UTMB setting would mirror those suggested by other researchers—that early introduction to correctional care rotations, prior to matriculation and longitudinally over the course of pre-clinical years, can help assuage these anxieties (Alemagno et al. 2004; Brooker et al. 2018). Importantly, this study stands out in providing a narrative where fears are overcome not only by exposure to the carceral environment, but also the learners’ reimagination of the incarcerated “other” as a positive outcome of performing a correctional care rotation.

Reflective of other studies (Alemagno et al. 2004; Kuthy et al. 2007; Brooker et al. 2018) and the rationale behind greater AMC involvement in correctional care (Raimer and Stobo 2004; Wakeman and Rich 2010; Pelletier 2015; Trestman et al. 2015), our findings suggest that correctional care training—singular among other traditional care settings—provides a unique opportunity to engage with the personhood of the patient by challenging prevailing stereotypes of the incarcerated and potentially other vulnerable communities. Here, we find some synergy with the aims of AMCs to create clinical experiences for medical learners that foster favorable attitudes to providing health care in prisons and reduce stigma towards those who are in custody or recently released (Faulkner and McCurdy 2000; Littlewood et al. 2005; Wakeman and Rich 2010; Min et al. 2012; Brooker et al. 2018; Candamo et al. 2018).

However, our study provides an important caveat to the previously reported positive outcomes of other studies in correctional health settings (Applebaum et al. 2002; Alemagno et al. 2004; Weiskopf 2005; Dhawan et al. 2007; Haley et al. 2009; Rich et al. 2012; Filek et al. 2013; Diaz et al. 2014; Bouchaud and Swan 2017; Simon et al. 2017; van de Mortel et al. 2017; Brooker et al. 2018; Candamo et al. 2018). Our data suggests a more cautionary approach may be required before immersing medical trainees (or their proctors) in correctional healthcare settings.

In a previous publication related to this data set, we identified how the provision/withholding of healthcare to and from persons who are incarcerated plays a major role in disciplining incarcerated bodies into becoming compliant medical patients and research subjects, complacent with and even grateful for delayed care, delivered sometimes below the standard best practices (Glenn et al. 2020). Here we sought to evaluate the formal and informal curriculum and its impact on the medical learner’s experience. The learning environment within a correctional care setting—characterized by institutional, organizational, and interpersonal elements of care—may substantiate broader concerns of a “hidden curriculum” that has led to spirited debate in medical education in recent years (Martimianakis et al. 2015; Martimianakis and Hafferty 2016; Lawrence et al. 2018). Beyond merely combating negative stereotypes of the incarcerated, medical trainees were susceptible to routine violations of the personhood of incarcerated patients while caught between dual loyalties to the goals of medicine and the goals of punishment by the state. Furthermore, the predominant negative attitudes of seasoned nurses and attending physicians witnessed by medical learners give us reason to suspect that the positive attitude changes of trainees may be difficult to sustain over time (Brazeau et al. 2010; Higashi et al. 2013; Baird et al. 2016).

As Kuthy and colleagues point out, “effective clinical educational programs need to go beyond mere minimum exposure to various populations” (Kuthy et al. 2007). In this study setting, the positive experiences of medical trainees most likely result from their own self-reflection and *in spite* of the learning environment and its prevailing culture of low prioritization and neglect. It is also apparent that although there were glimpses of potential positive transformation, most trainees left this environment with a lack of insight into the ethical and moral dimensions of training on the vulnerable. Participants in this study often espoused a clear interest in a correctional care training environment for its *instrumental value* in allowing them greater exposure to advanced pathology and disease uncommon in the general population. In exploring this, our study importantly provides some “moral” bounds to this perceived benefit—both among medical learners and more broadly within the academic medical community. This study suggests that the draw of advanced pathology for medical learners is precisely the result of trainees’ poor understanding of the social determinants of disease that the incarcerated face as they relate to dire health outcomes— the very health inequities correctional care proponents seek to redress. Another problematic pull toward correctional care for trainees noted here was the ability “to do more,” which implicates participants’ medical teams that allow trainees to practice on the incarcerated patients more than they would free world patients—an overt and subconscious perpetuation of bias toward incarcerated patients as less deserving of care. Participants’ inability to recognize how incarcerated patients’ autonomy is often ignored by virtue of their vulnerable position due to incarceration suggests that mere exposure to this environment does not yield greater insight.

Medical trainees enter their learning environments with their own biases and prejudices (Hester 2016) redolent of those held in society at large, but negative perceptions of incarcerated individuals risk being cemented without recourse to a structured curriculum that deconstructs such perceptions. Our respondents indicate that this is particularly needed for faculty preceptors, largely responsible for the hidden curriculum that further perpetuates a sense that the incarcerated are less deserving of care (Brazeau et al. 2010; Higashi et al. 2013; Baird et al. 2016). As a study by Dhawan and colleagues and another study by Restum point out, the belief that incarcerated patients are less deserving of care often compromises the quality of care (Restum 2005; Dhawan et al. 2007). Our study substantiates this fear, with medical learners pointing out how such bias among attending physicians and senior members of the medical team was not only prevalent but provided the scaffolding upon which a culture of low prioritization and neglect was established, leading to delayed diagnoses and treatment of incarcerated patients. As in other studies, participants were able to identify structural constraints of the correctional environment as leading to delays in care, often labeled by other researchers as “the nature of the setting” (Weiskopf 2005). However, our study findings deepen our understanding of how such elements—the built environment, institutional policies, and a culture of neglect—are inextricably linked in the prison health complex.

This study is one of the few that uses primary source data to characterize dual loyalty in correctional care settings (Pont et al. 2012)—the unique moral and ethical challenges medical learners face in being torn between the goals of medicine as rehabilitative and the emphasis of correctional systems on punishment. The published literature on the experiences of trainees in such environments only touches upon this notion (Alemagno et al. 2004; Haley et al. 2009; Sufrin et al. 2012). A decade ago, Haley and colleagues convened seasoned correctional medical professionals to comment and provide suggestions for curricula that engender correctional care competency among trainees (Haley et al. 2009). Tellingly, participants’ suggestions implied how these providers dealt with the question of dual loyalty in providing correctional care, recommending that trainees learn to “negotiate the provision of health care with security needs of the institution,” “understand the security mission as a means to enforce punishment and provide safety to inmates and staff,” and that a future correctional care provider should be able to create a “therapeutic alliance between provider and patient while maintaining boundaries without risking safety or security” (Haley et al. 2009).

The inability to navigate this ethical dimension among seasoned correctional care providers supports our study findings that trainees would also struggle with these issues, and that exposure alone—even after years of experience as many of the physicians and nurses in the Haley study and at the TDC Hospital possess—does little to forge a moral compass in addressing such professional conflicts. Given the recent backlash within the academic medical community in the US where physicians were solicited in *JAMA* to apply to work for a US Immigration and Customs Enforcement detention center with the caveat that applicants be “philosophically committed to the objectives of the facility” (Mishori 2019), more conscious effort should inform correctional care curricula that taps into the immense learning opportunities for ethically appropriate care that such environments afford. Whereas most educators have historically advocated for structured learning around the diseases unique to correctional populations, they have often left the ethics of correctional care to be acquired through something akin to “osmosis” by mere exposure to the incarcerated (Kaufman et al. 1979; Alemagno et al. 2004; Raimer and Stobo 2004; Weiskopf 2005; Haley et al. 2009; Wakeman and Rich 2010; Pelletier 2015; Trestman et al. 2015; Bouchaud and Swan 2017). Our study findings suggest that addressing health disparities among vulnerable populations such as those who are imprisoned would be better served if this “hierarchy” of competencies was reversed.

Therefore, we highlight some important implications of this study. Medical training programs that intend for some proportion of trainees to work with the incarcerated should consider a multi-stage orientation to the correctional care service. This could include more information prior to matriculation, training on vulnerable populations and structural competency (Metzl and Hansen 2014; Kasper et al. 2016), structured experiences in pre-clinical years (Littlewood et al. 2005; Filek et al. 2013), and ensuring that all clinical students receive such training prior to seeing patients on the wards. There should be time provided for critical reflection while trainees are on the wards that focus on the ethics of providing care in this environment, which can be rooted in the traditions of principlism, casuistry, and virtue ethics. Given the evidence for experiences in early medical education that can foster social responsiveness (Littlewood et al. 2005), pre-clinical years may also feature activities such as problem-based learning and patient interview interventions (Batt-Rawden et al. 2013). However, a recent intervention using simulation and patient interviews to orient nursing students to a correctional care environment leaves much to be desired, as this particular intervention seems to *perpetuate* negative stereotypes of justice-involved persons instead of addressing them (Diaz et al. 2014). Such poorly constructed interventions should impress upon educators the need to carefully plan and design interventions with appropriate and meaningful pre- and post-intervention outcomes of interest (e.g., empathy as opposed to security/safety) (Littlewood et al. 2005; Batt-Rawden et al. 2013).

However, interventions for medical trainees are only part of the solution. There is an obvious need for more intensive, in-service training for attending physicians, nursing faculty and staff, and auxiliary staff who also work in correctional settings. AMC administrators may consider a program for correctional care faculty and staff similar to that recommended for medical trainees that is provided during an orientation period prior to full-time work in correctional care. Staff and faculty should also have required mentorship training. An institution may find at its disposal a cadre of health professionals—who hail from different pedagogical training such as the medical humanities, clinical ethics, bioethics, social work, care management—who could be integrated to systematize opportunities for education on ethical clinical decision making.

This study is not without limitations. We must note that in the intervening years since this data was collected, UTMB has indeed implemented some of what is recommended: earlier engagement with medical trainees about the prospect of working with incarcerated populations as well as more timely orientation—involving elements of vulnerable populations and ethics training—to the correctional care service. A cross-sectional component to quantify attitudes about care for incarcerated patients among a much greater number of medical trainees through use of validated surveys would strengthen this project. In addition, the above implications for in-service training could be better informed by formative research not only among medical learners but also among faculty and staff working with incarcerated patients that follows the current study design, with an additional, quantitative component to assess attitudes toward the incarcerated. More systematic studies utilizing root cause analysis to evaluate institutional policies as they relate to delays in care and/or poor outcomes in incarcerated populations compared to free world patients would also provide valuable insight into developing and implementing training and curricula for correctional care providers and medical trainees.
